# Supplementary material for: Camostat mesilate inhibits pro-inflammatory cytokine secretion and improves cell viability by regulating MFGE8 and HMGN1 in lipopolysaccharide-stimulated DF-1 chicken embryo fibroblasts
Source: PeerJ. 2021 Aug 26;9:e12053. doi: 10.7717/peerj.12053 (PMC8403478; doi:10.7717/peerj.12053)
Supplement: Supplemental Information 1 [file peerj-09-12053-s001.docx]

**TMT analysis**

**Sample preparation**

Cells were lysed using the protein lysis buffer (7M Urea/2M Thiourea/4% SDS/40 mM Tris-HCl, pH 8.5/1mM PMSF/2mM EDTA). After the addition of DTT (final concentration: 10 mM), cell lysates were sonicated for 15 min on ice and then centrifuged at 13,000×g and 4°C for 20 min. The supernatants were transferred to new centrifuge tubes and then mixed with four volumes of -20 °C prechilled acetone. The mixtures were kept overnight at -20°C. After centrifuged for 15 min at the conditions of 8,000×g and 4°C, protein pellets were collected, dried in the air and re-dissolved in 8 M urea/100 mM triethylammonium bicarbonate (TEAB) (pH 8.0) solution. Next, proteins were reacted with 10 mM DTT for 30 min at 56°C and 55 mM iodoacetamide (IAM) for 30 min at room temperature in the dark. Next, protein concentration was determined using the Bradford method. After diluted 5-fold with 100 mM TEAB, proteins (100μg/sample) were digested overnight with trypsin at an enzyme-protein ratio of 1:50 (w/w) at 37°C. The peptide segments post enzymolysis were desalted using C18 columns and then dried using the vacuum freezing method.

**TMT labeling**

Next, the dried peptides were redissolved in TEAB solution, and then labeled using the TMT label reagent. Next, the reactions were quenched using 2.7% aqueous [aqua] ammonia. Subsequently, equal amounts of TMT-labeled samples were pooled, desalted and freeze-dried.

**Peptide fractionation**

Next, labeled peptide powders were dissolved in the solution (PH=10) containing 2% acetonitrile and 98% H_2_O. After centrifugation, supernatants were fractionated on L-3000 high-performance liquid chromatography (HPLC) system. Distillate fractions were collected per minute and merged into 12 fractions.

**[Liquid](https://cn.bing.com/dict/clientsearch?mkt=zh-CN&setLang=zh&form=BDVEHC&ClientVer=BDDTV3.5.1.4320&q=%E6%B6%B2%E7%9B%B8%E8%89%B2%E8%B0%B1-%E8%B4%A8%E8%B0%B1%E8%81%94%E7%94%A8%E5%88%86%E6%9E%90)** **[chromatography](https://cn.bing.com/dict/clientsearch?mkt=zh-CN&setLang=zh&form=BDVEHC&ClientVer=BDDTV3.5.1.4320&q=%E6%B6%B2%E7%9B%B8%E8%89%B2%E8%B0%B1-%E8%B4%A8%E8%B0%B1%E8%81%94%E7%94%A8%E5%88%86%E6%9E%90)****[-](https://cn.bing.com/dict/clientsearch?mkt=zh-CN&setLang=zh&form=BDVEHC&ClientVer=BDDTV3.5.1.4320&q=%E6%B6%B2%E7%9B%B8%E8%89%B2%E8%B0%B1-%E8%B4%A8%E8%B0%B1%E8%81%94%E7%94%A8%E5%88%86%E6%9E%90)****[mass](https://cn.bing.com/dict/clientsearch?mkt=zh-CN&setLang=zh&form=BDVEHC&ClientVer=BDDTV3.5.1.4320&q=%E6%B6%B2%E7%9B%B8%E8%89%B2%E8%B0%B1-%E8%B4%A8%E8%B0%B1%E8%81%94%E7%94%A8%E5%88%86%E6%9E%90)** **[spectrometry](https://cn.bing.com/dict/clientsearch?mkt=zh-CN&setLang=zh&form=BDVEHC&ClientVer=BDDTV3.5.1.4320&q=%E6%B6%B2%E7%9B%B8%E8%89%B2%E8%B0%B1-%E8%B4%A8%E8%B0%B1%E8%81%94%E7%94%A8%E5%88%86%E6%9E%90) analysis**

Next, the samples were freeze-dried and re-dissolved in A solution composed of 0.1% formic acid and 100% H_2_O. After centrifugation, supernatants containing proteins were analyzed using EASY-nLCTM 1200 Ultra High Performance Liquid Chromatography (UHPLC, Thermo Fisher Scientific) and Q Exactive HF-X Mass Spectrometer (Thermo Fisher Scientific).

**Protein identification**

Proteins were identified using Proteome Discoverer 2.2 (Thermo Fisher Scientific). The retrieval arguments of Proteome Discoverer 2.2 were presented as blow.

| **Item** | **Value** |
| --- | --- |
| **Type of search** | Reporter Quantification(TMT 10 plex) |
| **Enzyme** | Trypsin |
| **Instrument** | Thermo Q Exactive™ HFX |
| **Max.Missed Cleavage Sites** | 2 |
| **Precursor Mass Tolerance** | 10 ppm |
| **Fragment Mass Tolerance** | 0.02 Da |
| **Dynamic Modification** | Oxidation/+15.995 Da (M) and TMT 10plex/+229.163 Da (K) |
| **N-Terminal Modification** | Acetyl/+42.011 Da (N-Terminal) and TMT 10plex/+229.163 Da (N-Terminal) |
| **Static Modification** | Carbamidomethyl/+57.021 Da (C) |

**Protein quantification**

Proteins were quantified using Maxquant software. Differences between groups were analyzed using Student’s t test. P values obtained from Student’s t test were corrected through false discovery rate (FDR). The values after FDR correction were termed as Q value.

**Differential expression analysis**

Proteins (fold change≥1.2 or ≤0.83 and Q value≤0.05) were considered to be significantly differentially expressed.
