# Supplementary figures and images for: Camostat mesilate inhibits pro-inflammatory cytokine secretion and improves cell viability by regulating MFGE8 and HMGN1 in lipopolysaccharide-stimulated DF-1 chicken embryo fibroblasts
Source: PeerJ. 2021 Aug 26;9:e12053. doi: 10.7717/peerj.12053 (PMC8403478; doi:10.7717/peerj.12053)

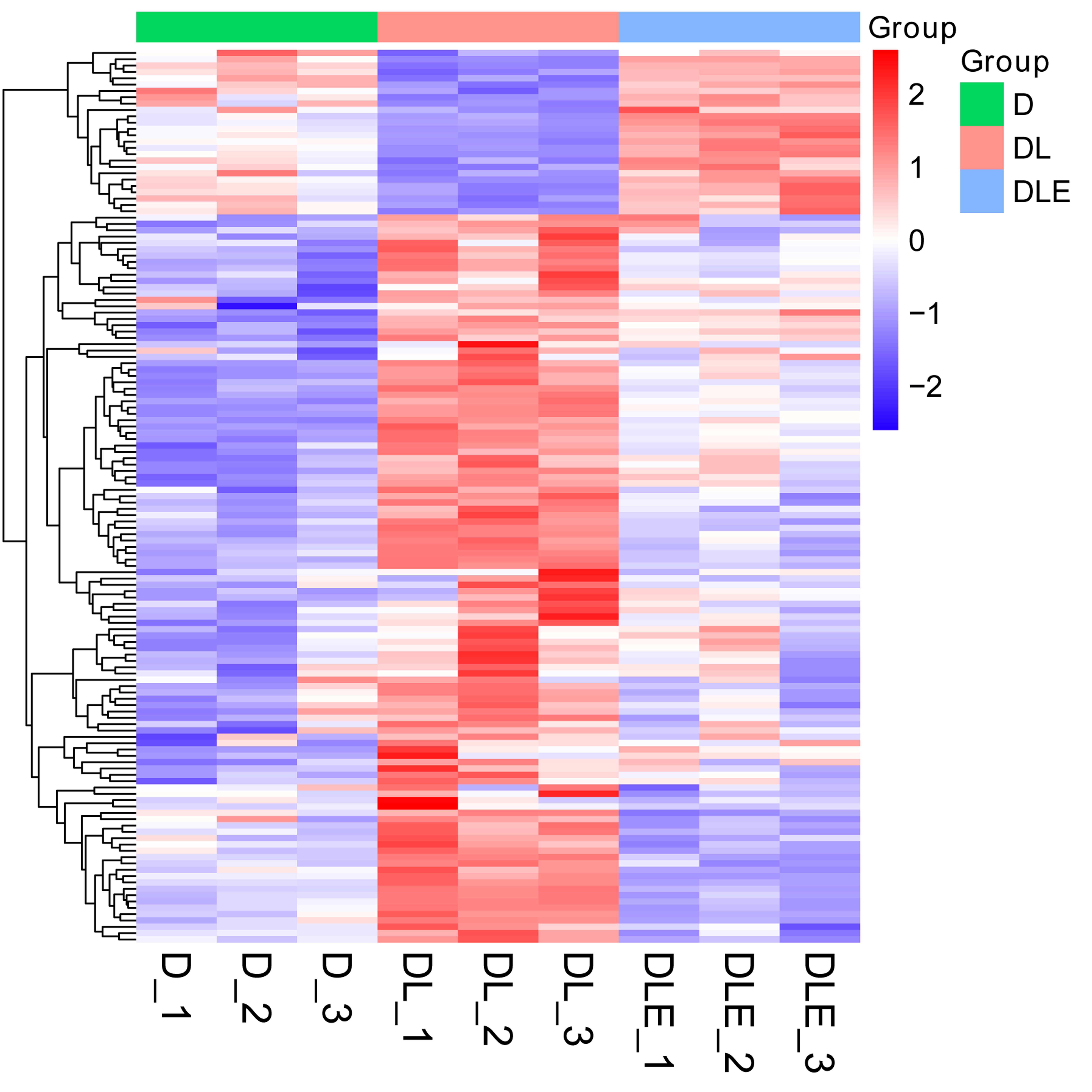

Supplement: Supplemental Information 2 [file peerj-09-12053-s002.png]

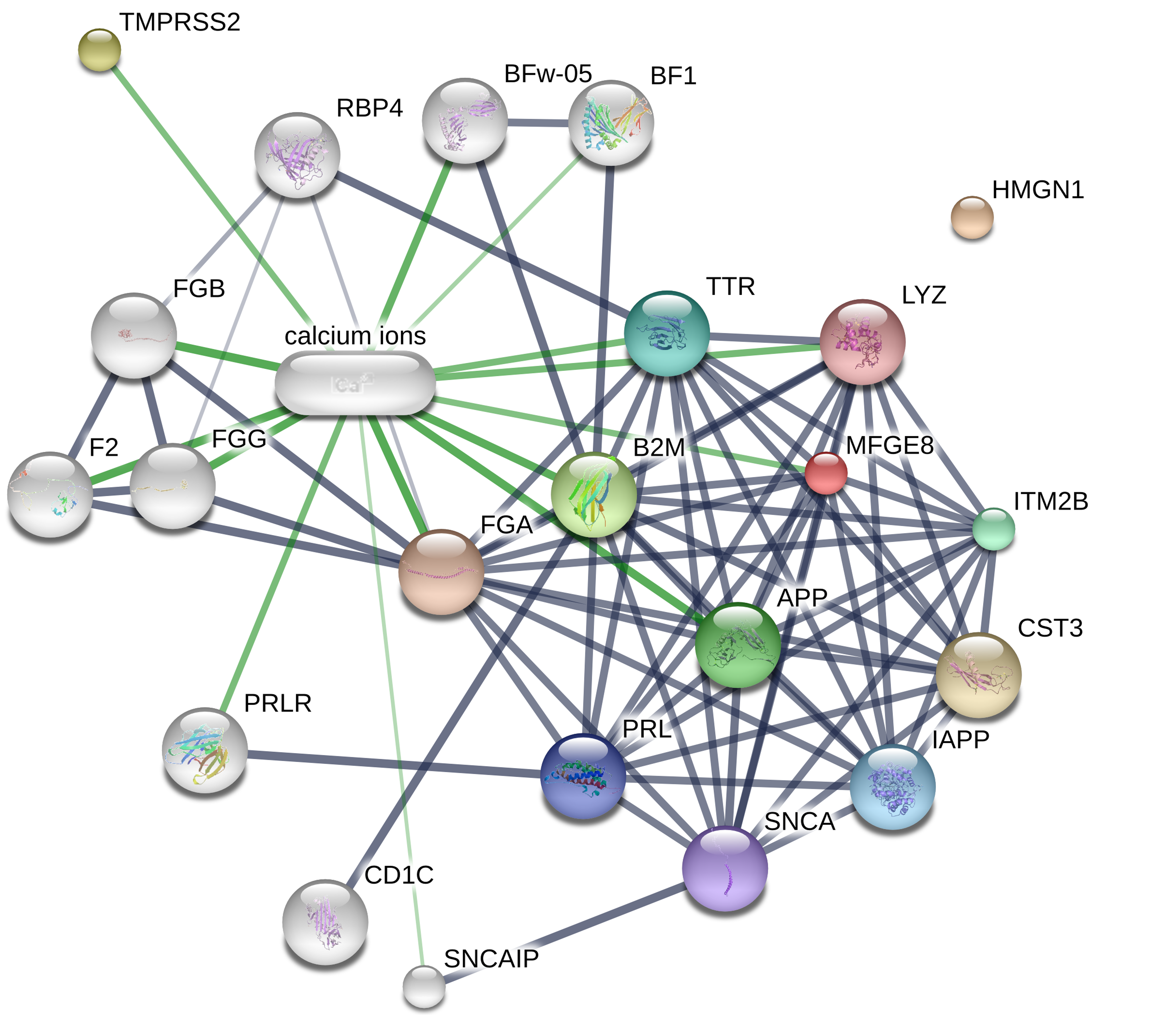

Supplement: Supplemental Information 3 [file peerj-09-12053-s003.png]

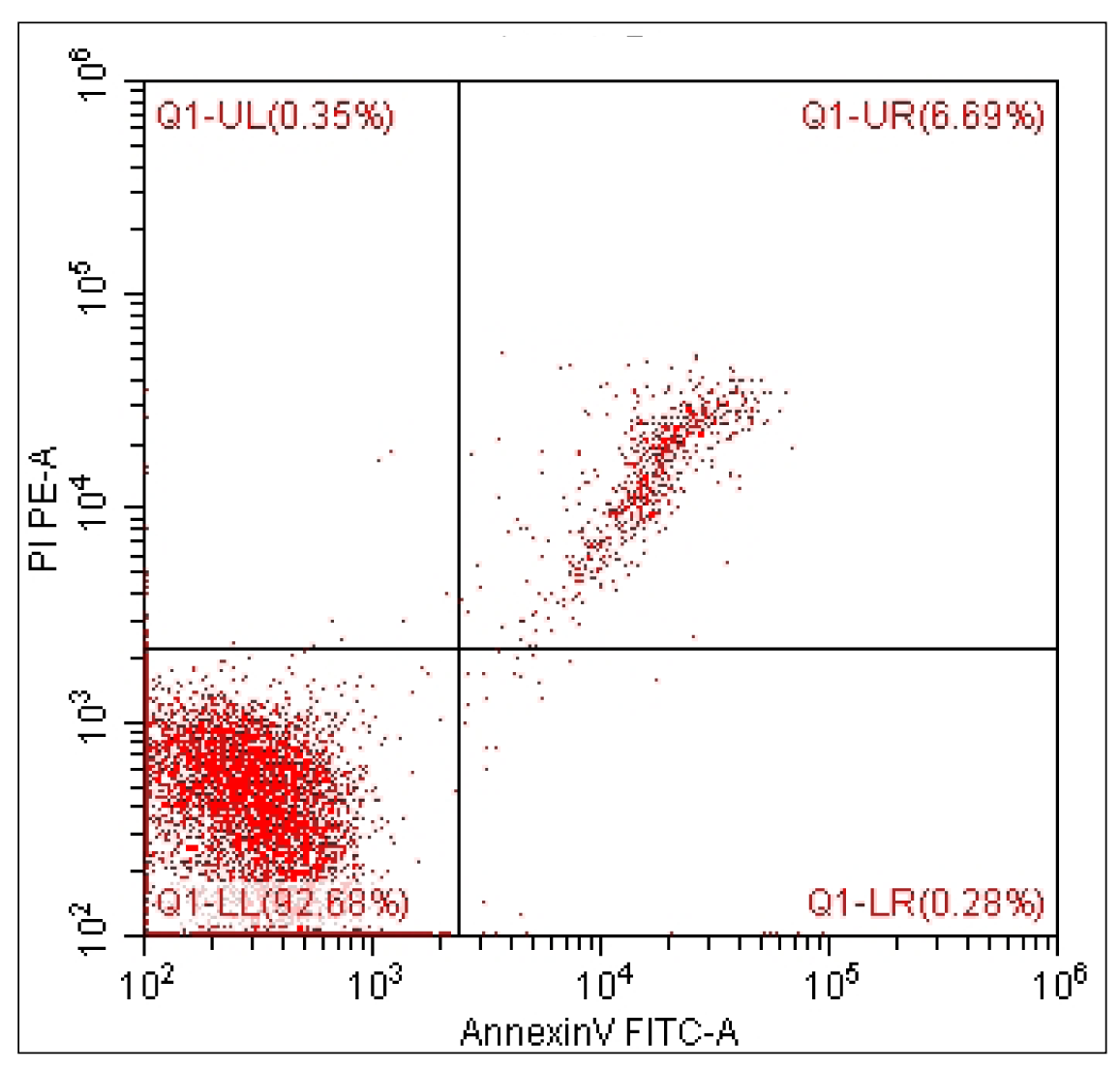

Supplement: Supplemental Information 5 [file peerj-09-12053-s005.zip › raw data/cell apoptsosis/control.tif]

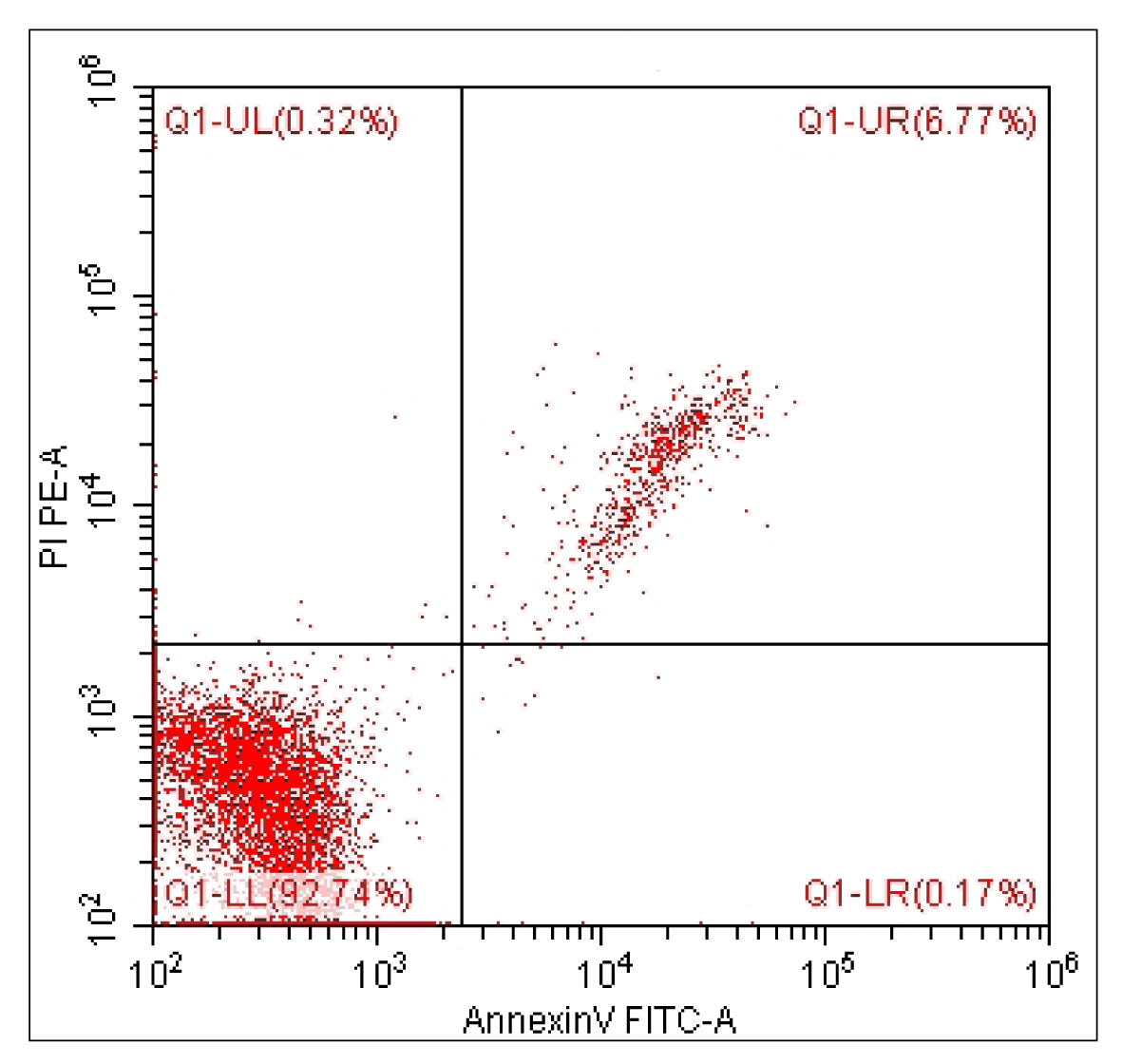

Supplement: Supplemental Information 5 [file peerj-09-12053-s005.zip › raw data/cell apoptsosis/LPS+CM+si-HMGN1#2.tif]

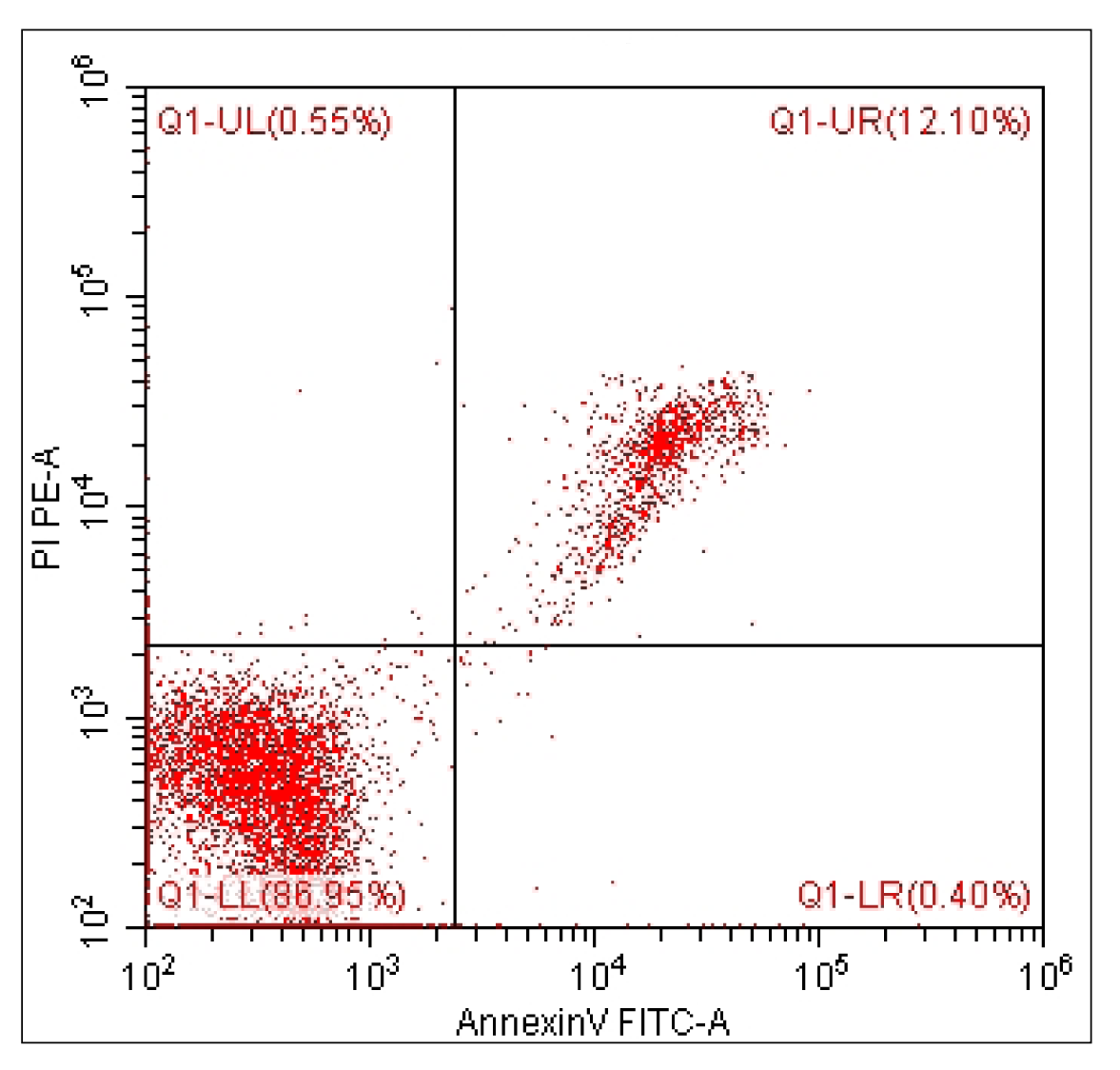

Supplement: Supplemental Information 5 [file peerj-09-12053-s005.zip › raw data/cell apoptsosis/LPS+CM+si-MFGE8#1.tif]

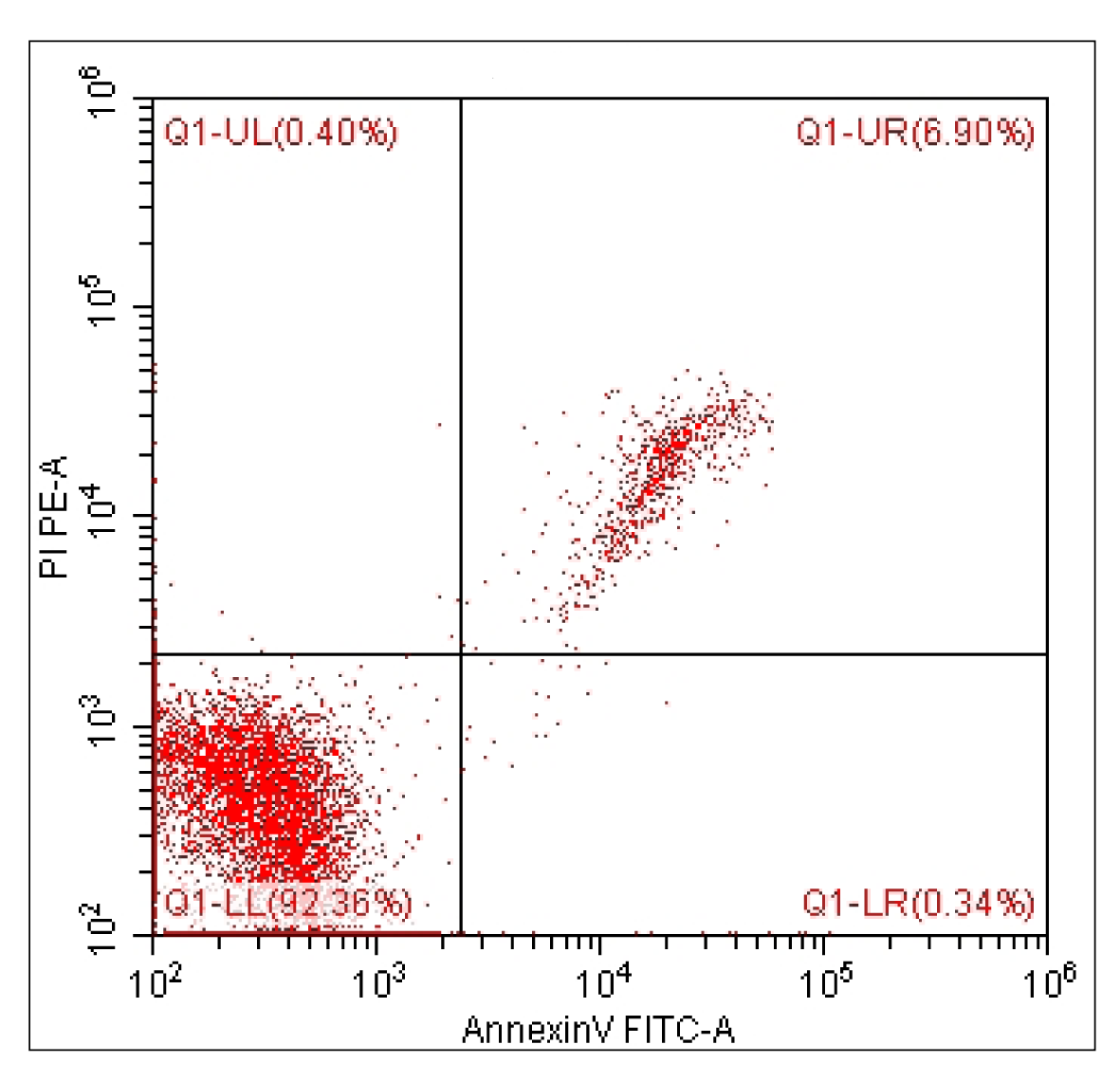

Supplement: Supplemental Information 5 [file peerj-09-12053-s005.zip › raw data/cell apoptsosis/LPS+CM+si-NC.tif]

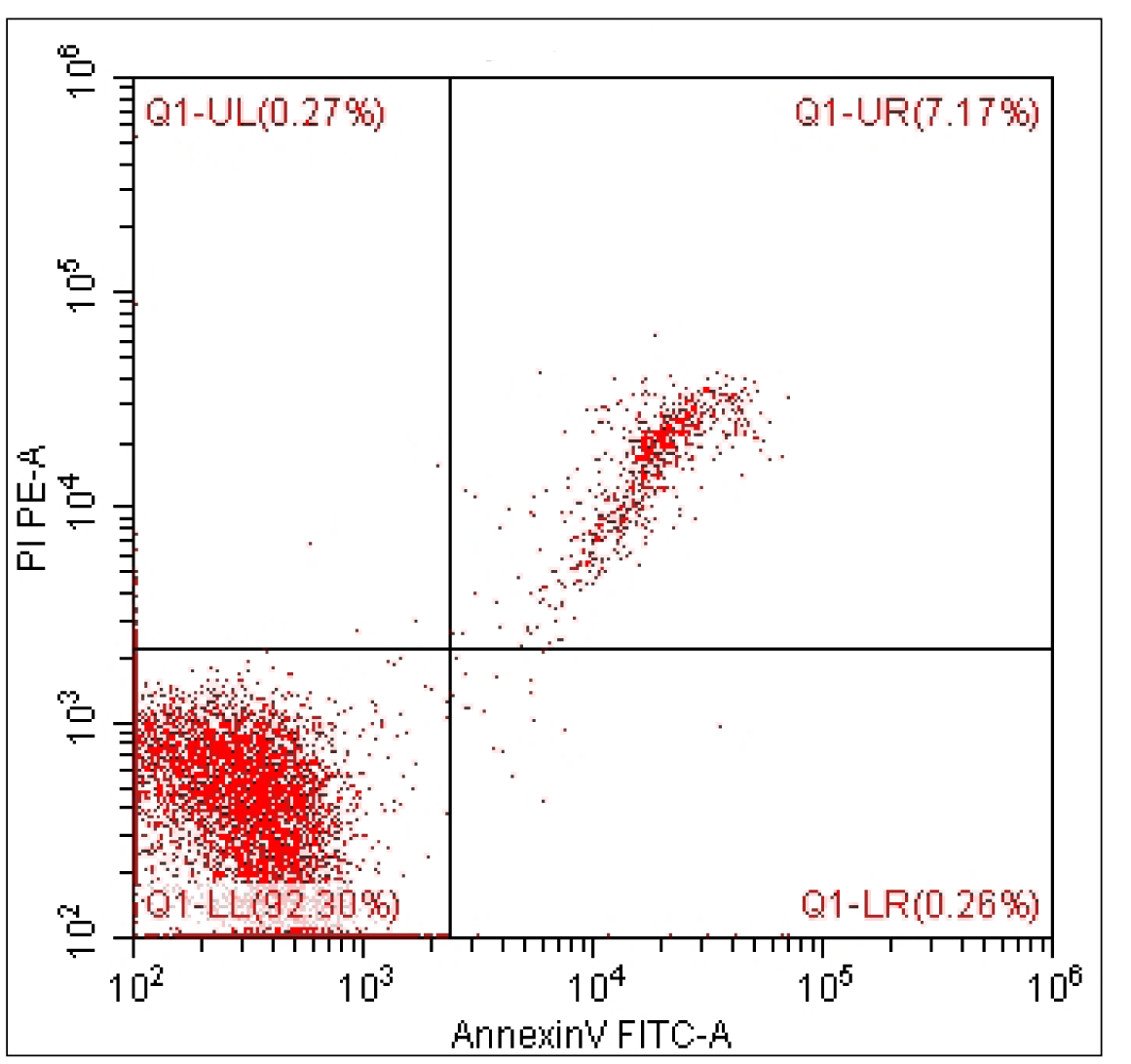

Supplement: Supplemental Information 5 [file peerj-09-12053-s005.zip › raw data/cell apoptsosis/LPS+CM.tif]

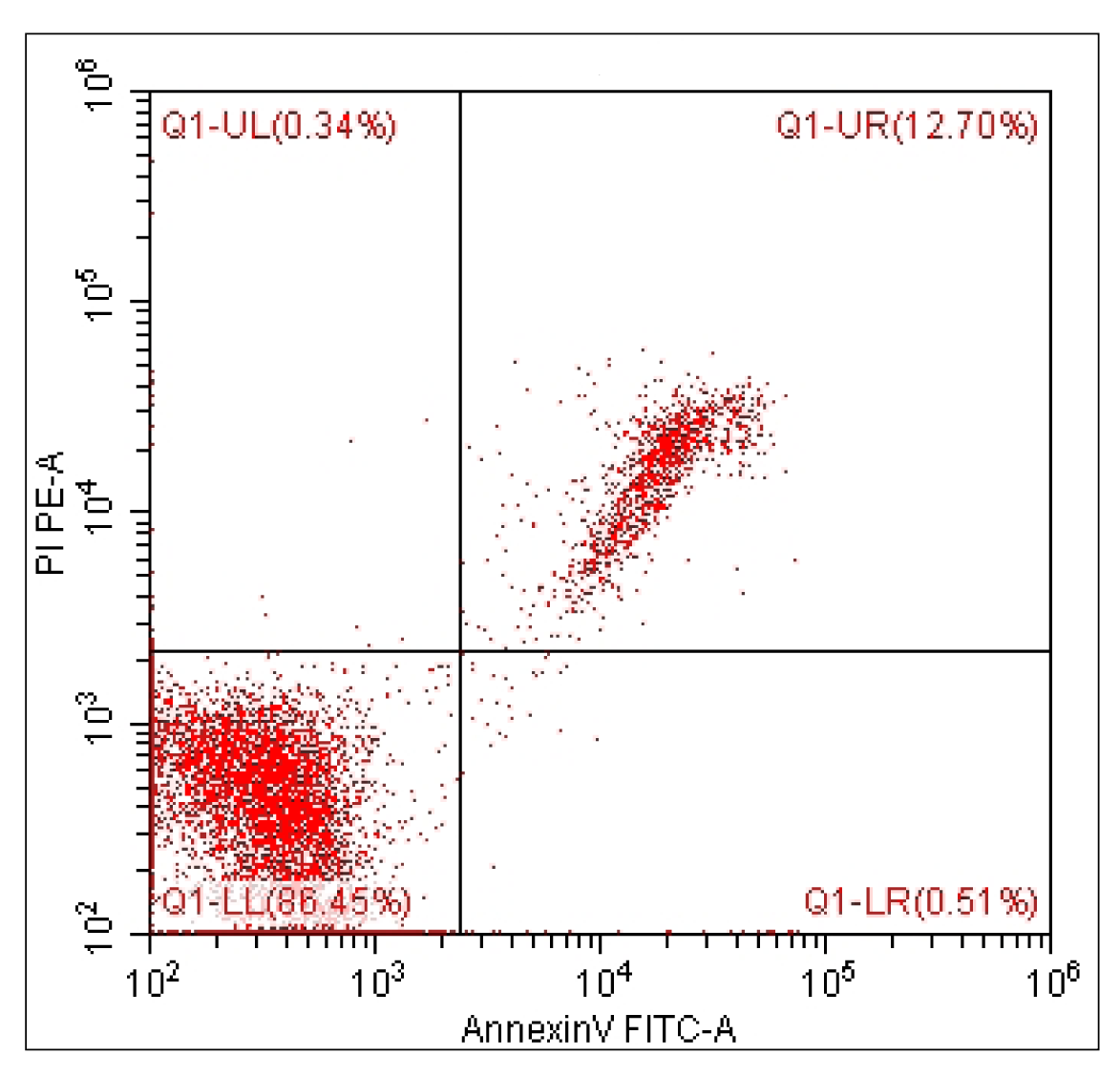

Supplement: Supplemental Information 5 [file peerj-09-12053-s005.zip › raw data/cell apoptsosis/LPS.tif]
